# A Study on the Diagnostic Elements of Cold-Heat Pattern Identification by Korean Medicine Doctors: Association with Objective and Subjective Body Temperature

**DOI:** 10.1155/2017/7593056

**Published:** 2017-12-13

**Authors:** Young Joo Park, Ji-Ho Nam, Mi Hong Yim, Honggie Kim, Jong Yeol Kim

**Affiliations:** ^1^KM Fundamental Research Division, Korea Institute of Oriental Medicine (KIOM), 1672 Yuseong-daero, Yuseong-gu, Daejeon, Republic of Korea; ^2^Korean Medicine Life Science, University of Science and Technology, 217 Gajeong-ro, Yuseong-gu, Daejeon, Republic of Korea; ^3^Medizen Humancare Inc., 20F Keungil Tower, 223 Teheran-ro, Seoul, Republic of Korea; ^4^Department of Information and Statistics, Chungnam National University, 99 Daehak-ro, Yuseong-gu, Daejeon, Republic of Korea

## Abstract

Although the Cold-Heat Pattern is the most important diagnostic factor in Traditional Korean Medicine (TKM), its relationship to body temperature and subjective temperature has not been clearly revealed. In this study, based on clinical data from 551 patients, we classified patients treated with herbal medicines into a Cold-prescription group (CG) and a Heat-prescription group (HG), and we compared the ordinary symptoms between the two groups. Subjective body temperature was higher in the HG than in the CG (OR = 1.68, *p* < 0.01) and digestive ability was better in the HG than in the CG (expert's questionnaire, OR = 1.91, *p* < 0.001). However, objectively measured body temperature did not show any significant difference between the HG and CG in both gender groups (*p* = 0.383 and 0.181 for males and females, resp.). Our study suggests that the subjective body temperature and digestive ability may be the principal diagnostic elements of Cold-Heat Pattern identification by Korean Medicine Doctors. These findings may contribute to the investigation of an objective method to measure the Cold-Heat Pattern.

## 1. Introduction

As a patient-specific medicine, Traditional Korean Medicine (TKM), similar to Traditional Chinese Medicine (TCM), is characterized by pattern identification, including the Cold Pattern and the Heat Pattern. TKM and TCM doctors treat patients based on pattern identification reflecting personal body characteristics; thus, patients suffering from the same disease, such as stroke, are prescribed different herbal drugs if they present with a different pattern [1].

The Eight Principle Pattern Identifications are described in the “Diagnosis” part of the WHO International Standard Terminologies on Traditional Medicine in the Western Pacific Region, such as Exterior-Interior Pattern, Cold-Heat Pattern, Deficiency-Excess Pattern, and Yin-Yang Pattern [2]. Among the 8 patterns, the concept of the Cold-Heat Pattern is more concrete and easily acceptable than the other patterns [3].

Many studies using the concept of the Cold-Heat Pattern had been conducted such as the correlation between the Cold-Heat Pattern and diseases [4], the diagnosis consistency of the Cold-Heat Pattern [5], and the development of an optimized Cold-Heat Pattern questionnaire [6]. Diagnostic factors to determine the Cold-Heat Pattern such as the acoustic index [7], respiratory index [8], and bodily differences [9] have also been studied. However, surprisingly, the relationship between body temperature and the Cold-Heat Pattern has not been studied.

Sasang Constitutional Medicine (SCM) classifies people into four constitutional types, Tae-Eum (TE), So-Eum (SE), So-Yang (SY), and Tae-Yang (TY). SCM practitioners also treat patients with different herbal drugs according to 4 types. And for the treatment of diseases, every constitutional type is classified according to the Cold-Heat Pattern, which is related to the Exterior-Interior Pattern [10]. Thus, the Cold-Heat Pattern is the most important pattern identification for SCM.

Diagnoses of the Cold-Heat Pattern had been performed with various factors including the ordinary symptoms, the color of face and urine, and pulse characteristics. Recently several studies about diagnostic factors of the Cold-Heat Pattern for each constitutional type had been performed [11–13]. However, the relationship between body temperature and the Cold-Heat Pattern has not been studied so far.

In this study, we tried to identify a crucial diagnostic factor of the Cold-Heat Pattern. For this study, we used the measurement data of body temperature and the questionnaire for patients and doctors to record the ordinary symptoms, which were systematically collected and managed in the Korea Constitutional Multicenter Bank (KCMB).

## 2. Methods

### 2.1. Data Selection and Classification

Sasang medicine prescription cases collected by the KCMB were used. Data collected from 2013 to 2015 were included. Out of 915 subjects treated with Sasang medicine at Korean Medicine Clinics, a total of 551 TE, SE, and SY type subjects were included. They were prescribed one of the following: Taeumjowi-tang, Choweseuncheng-tang, Galgeunhaegi-tang, Yuldahanso-tang, Jeokbaekhaogwanjung-tang, Leejung-tang, Hyangsayangwi-tang, Palmulgunja-tang, Bojungikgi-tang, Hyungbangsabaek-san, Hyeongbangdojeok-san, Hyeongbangjihwang-tang, or Yanggyeoksanhwa-tang. The Tae-Yang type was excluded due to lack of subjects.

Each Sasang prescription was further classified into either the Cold- or Heat-prescription (Table 1). According to the prescription, 551 patients were classified into a CG and a HG.

### 2.2. Questionnaires and Ordering

We utilized 2 types of questionnaire, one for experts and another for patients. In expert's questionnaire, the doctor consulted with the patient and recorded the answers. In the patient's questionnaire, the patient recorded their own answers. The answers to the questionnaire consisted of category-type data, and we ordered the answers by degree. Five-point scale was employed in the expert's questionnaire: 1 point indicated very little tendency, 2 points indicated little tendency, 3 points indicated normal tendency, 4 points indicated large tendency, and 5 points indicated very large tendency. But, 3-point scale was employed in the patient's questionnaire: 1 point indicated little tendency, 2 points indicated normal tendency, and 3 points indicated a large tendency. The questionnaire and the answers are listed in Table 2.

### 2.3. Statistical Analysis

We analyzed the associations of ordinary symptoms between the HG and CG. Based on the preliminary analysis, difference was seen in the results on questions related to the subjective body temperature and digestive ability among two groups.

An ordered logit model, which is typically used to model ordered categorical responses on independent variables, was used to analyze the ordinary symptoms based on Cold-Heat prescription group, sex, and Sasang constitution. Among males and females, the female group was selected as the base category because they feel colder and have a weaker digestive function. SE type was also selected as the base category due to the same reasons [14, 15].

Correspondence analysis was applied to provide a visual explanation of two variables, subjective body temperature and digestive ability, especially in the expert's questionnaire. In patient's questionnaire data, correspondence analysis may not be useful since there are only 3 categories.

Correspondence analysis is a dimension reduction technique used in explanatory studies of contingency tables, which does not indicate any statistical significance but rather demonstrates the relationship between the categorical variables in a two-dimensional plane. Principal component analysis is used to perform a similar assessment of a set of continuous variables. SPSS 20 (IBM Corp., Armonk, NY) was used for all statistical computations, and statistical significance was established at *p* < 0.05.

### 2.4. Institutional Review Board (IRB)

This study was approved by the IRB of the Korea Institute of Oriental Medicine (I-1210/002-002-03).

## 3. Results

### 3.1. General Characteristics of the Subjects

The Sasang medicine prescription cases included information on age, height, weight, body mass index (BMI), and measured temperature according to sex and prescription (Table 3). There were significant differences between the HG and CG in weight and BMI for females, but no significant difference in body temperature (*p* = 0.383 and 0.181 for males and females, resp.). And no other characteristics show any difference between the HG and CG.

### 3.2. Analysis of Ordinary Symptoms

Table 4 shows the results for the ordered logit model estimation. There were 2 types of questionnaires (expert and patient); each questionnaire composed of questions related to subjective temperature and digestion. CG, females, and SE type were set as the base categories.

#### 3.2.1. Results for the Expert's Questionnaire

The odds of the HG's subjective body temperature were 68% higher than those of the CG after adjusting for Sasang type and sex (OR: 1.68, *p* < 0.01). And the odds of the HG's digestive ability were 91% higher than those of the CG after adjusting for Sasang type and sex (OR: 1.91, *p* < 0.001). Although there was no significant association, HG tends to have more appetite (OR: 1.26, *p* = 0.169).

#### 3.2.2. Results for the Patient's Questionnaire

The odds of the HG's warmness in hands were 44% higher than those of the CG after adjusting for Sasang type and sex (OR: 1.44, *p* < 0.05). And the odds of the HG's digestive ability were 69% higher than those of CG after adjusting for Sasang type and sex (OR: 1.69, *p* < 0.01). In other ordinary symptoms, the odds of HG were larger than those of CG, but there was no significant association.

And there were also significant associations between sex and ordinary symptoms. In both expert's and patient's questionnaires, the subjective body temperature and the digestive ability of male tended to be higher than those of female. Among the 3 Sasang types, TE type and SY type tended to have higher subjective body temperature and better digestive ability than SE type.

#### 3.2.3. Association between Subjective Body Temperature and Digestive Ability in Expert's Questionnaire

**Figure 1:**
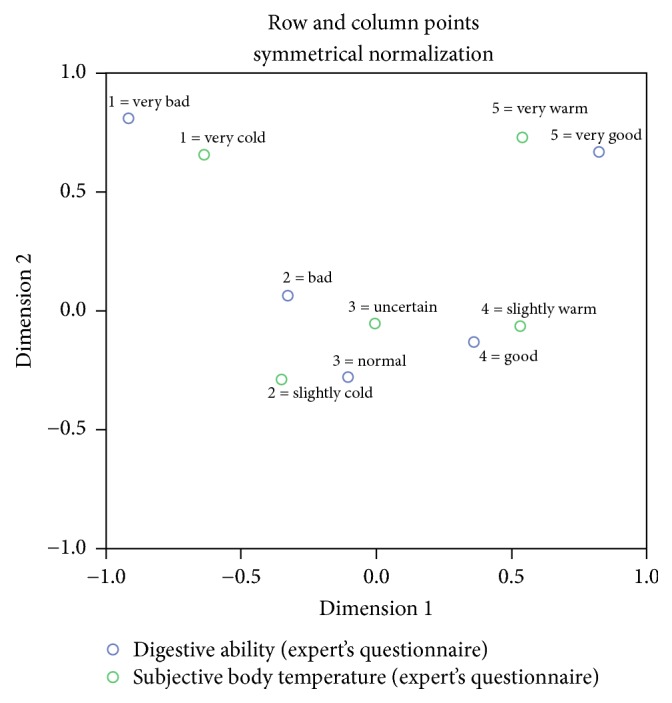
Correspondence analysis of digestive ability with subjective body temperature.

Furthermore, we explored the association of subjective body temperature with digestive ability using the correspondence analysis. Bad digestive ability was highly associated with a cold temperature, and good digestive ability was highly associated with a warm temperature (Figure 1).

## 4. Discussion

The main result of the current study was that the CG and HG were associated not with objectively measured body temperature, but with the warmness of hands in the patient's questionnaires. The expert's questionnaires also helped us find that the subjective body temperature of the HG was higher than that of CG.

However, unlike expectations, the measured body temperature of HG was not higher than that of CG. These results imply that the actual body temperature may not a diagnostic factor for doctors to decide the Cold- or Heat-prescriptions.

The other significant results give the possible explanation for the reason that subjective body temperature is a diagnostic factor of doctors to decide the Cold- or Heat-prescriptions. The CG and HG were also associated with digestive ability. Moreover, a close relationship between subjective body temperature and digestive ability was found through the correspondence analysis. Higher subjective body temperature was associated with better digestive ability. This finding suggests that digestive ability can strongly affect subjective body temperature and determine the doctor's decision for Cold- or Heat-prescription.

Subjective body temperature is experienced differently depending on a person's sensation, which has been found to be affected by fat distribution, BMI, and heart rate [16–18]. Wang et al. showed that hand temperature controls overall subjective body temperature. In a cold condition, the temperature of the fingers and hand gets consistently lower than that of other skin regions, whereas the core temperature remains steady. Heating the hand improved thermal comfort in the cold condition [19]. Another study found that cold hands and feet are more closely related to heritable factors than either sex differences or environmental influences [20]. The characteristics of subjective body temperature have been studied but not fully revealed yet.

Many consistent descriptions exist in classic TKM, such as “The Treasure of Oriental Medicine,” which states that a person who has a weak pulse and cold body will experience loss of appetite. According to “The Yellow Emperor's Canon Internal Medicine,” the Cold Pattern also causes diarrhea. “Danxi Xinfa Danxi's Mastery of Medicine” states that if the body becomes cold when eating food, digestion is inhibited. If the body becomes warmer, digestion is aided. These descriptions explain the influence of the Cold-Heat Pattern on digestion.

“Longevity & Life Preservation in Oriental Medicine,” the classic of SCM, also suggests an association of the Cold-Heat Pattern with digestion. For example, an SE type patient with a Cold Pattern shows symptoms related to a digestive disorder, and a colder stomach is associated with a worse digestive function [21]. Herbal drugs for the Cold Pattern of SE type have strong efficacy in strengthening digestive function. Generally, herbal drugs for the Cold Pattern, such as magnolol, have hot properties to activate the gastrointestinal tract [22].

Several studies also support the relationship of the Cold-Heat Pattern with digestion. Recently, Bae et al. studied the association of cold hypersensitivity in the hands and feet with functional dyspepsia [23]. This study suggested the association of the Cold-Heat Pattern with digestive function and stated that cold stimulation of the body could cause digestive diseases. Similar findings have been found in animal tests [24, 25].

However, most of these studies did not consider clinical prescriptions. In our study, using the clinical data, we found that subjective body temperature and warmness in hands were the decisive diagnostic factors of doctors' prescriptions according to the Cold-Heat Pattern, which was closely related to digestive ability.

## 5. Conclusions

In the present study, we found that subjective body temperature and warmness in hands, not measured body temperature, were the diagnostic factors of doctors to decide the Cold- or Heat-prescriptions and that subjective body temperature is closely related to digestive ability.

But here is a limitation that these results were based on only medical interviews or questionnaires. If our study is combined with other existing researches such as thermal sensation's association with brain waves [26] and brain activation acquired by MRI [27], the physiological meaning of the Cold-Heat Pattern could be more clearly interpreted.

## Figures and Tables

**Table 1:** Formula and classification [10].

Sasang type	Prescription type	Pattern Identification	Name of prescription	Males	Females
TE type	Cold-prescription	Exterior cold disease induced from the stomach duct affected by cold	Taeumjowi-tang	11	21
			Choweseuncheng-tang	17	28
	Heat-prescription	Interior febrile disease induced from the liver affected by heat	Galgeunhaegi-tang	12	10
			Yuldahanso-tang	38	63

SE type	Cold-prescription	Interior cold disease induced from the stomach affected by cold	Jeokbaekhaogwanjung-tang	2	9
			Gwankeibujalijung-tang	6	13
			Baekhaolijung-tang	1	2
			Osuyubujalijung-tang	0	1
			Hyangsayangwi-tang	3	6
	Heat-prescription	Exterior febrile disease induced from the kidney affected by heat	Palmulgunja-tang	32	41
			Bojungikgi-tang	3	0

SY type	Cold-prescription	Exterior cold disease induced from the spleen affected by cold	Hyungbangsabaek-san	9	16
			Hyeongbangdojeok-san	3	33
			Hyeongbangjihwang-tang	22	86
	Heat-prescription	Interior febrile disease induced from the stomach affected by heat	Yanggyeoksanhwa-tang	29	34

**Table 2:** Questionnaire of ordinary symptoms and answers.

	Ordinary symptom		Response
For expert	Subjective body temperature		1 = very cold; 2 = slightly cold; 3 = normal; 4 = slightly hot; 5 = slightly hot
	Sensitivity to cold		1 = very insensitive; 2 = insensitive; 3 = normal; 4 = sensitive; 5 = very sensitive
	Sensitivity to heat		1 = very insensitive; 2 = insensitive; 3 = normal; 4 = sensitive; 5 = very sensitive
	Digestive ability		1 = very bad; 2 = bad; 3 = normal; 4 = good; 5 = very good
	Appetite		1 = very bad; 2 = bad; 3 = normal; 4 = good; 5 = very good

	Ordinary symptom	Question	Response

For patient	Warmness in hands	Do your hands tend to be cold or warm?	1 = cold; 2 = normal or uncertain; 3 = warm
	Warmness in feet	Do your feet tend to be cold or warm?	1 = cold; 2 = normal or uncertain; 3 = warm
	Warmness in abdomen	Does your abdomen tend to be cold or warm?	1 = cold; 2 = normal or uncertain; 3 = warm
	Digestive ability	Do you have any discomfort due to indigestion?	1 = indigestion and discomfort; 2 = indigestion but no discomfort; 3 = good digestion
	Appetite	How is your usual appetite?	1 = poor; 2 = normal; 3 = good or very good
	Food portion	What is the average daily portion of each meal?	1 = small; 2 = medium; 3 = big or irregular
	Eating pace	What is your average eating pace?	1 = slow; 2 = normal; 3 = fast

**Table 3:** General characteristics of subjects according to Sasang type.

	Males			Females		
	CG	HG	*p* value	CG	HG	*p* value
*N*	74	114		215	148	

Age	43.41 ± 1.67	47.03 ± 1.51	0.118	45.40 ± 1.02	45.93 ± 1.29	0.745
Height (cm)	172.11 ± 0.77	171.27 ± 0.60	0.389	158.43 ± 0.39	159.16 ± 0.44	0.220
Weight (kg)	71.70 ± 1.21	70.86 ± 0.97	0.592	56.00 ± 0.58	59.85 ± 0.74	<0.001
BMI (kg/m^2^)	24.21 ± 0.39	24.13 ± 0.29	0.865	22.30 ± 0.22	23.63 ± 0.28	<0.001
Body temperature (°C)	36.42 ± 0.04	36.46 ± 0.02	0.383	36.57 ± 0.02	36.62 ± 0.02	0.181

Data are presented as the means ± standard error. *p* values were calculated by comparison the CG with HG using Student's *t*-test.

**Table 4:** Ordered logit model estimation results for association between the Cold- or Heat-prescription group and ordinary symptoms.

Type of questionnaire	Ordinary symptom	HG	OR	Males	OR	TE type	OR	SY type	OR
		B (95% CI)		B (95% CI)		B (95% CI)		B (95% CI)	
Expert	Subjective body temperature	0.52 (0.17~0.87)	1.68^*∗∗*^	1.25 (0.91~1.60)	3.51^*∗∗∗*^	0.92 (0.46~1.38)	2.51^*∗∗∗*^	0.99 (0.50~1.47)	2.68^*∗∗∗*^
	Sensitivity to cold	−0.29 (−0.61~0.04)	0.75	−1.06 (−1.40~−0.73)	0.35^*∗∗∗*^	−0.85 (−1.26~−0.43)	0.43^*∗∗∗*^	−0.75 (−1.17~−0.33)	0.47^*∗∗∗*^
	Sensitivity to heat	0.09 (−0.23~0.42)	1.10	−0.27 (−0.59~0.06)	0.77	0.36 (−0.05~0.77)	1.43	0.39 (−0.02~0.81)	1.48
	Digestive ability	0.65 (0.32~0.98)	1.91^*∗∗∗*^	0.77 (0.44~1.10)	2.17^*∗∗∗*^	0.47 (0.05~0.88)	1.59^*∗*^	0.64 (0.22~1.06)	1.89^*∗∗*^
	Appetite	0.23 (−0.10~0.56)	1.26	0.65 (0.32~0.98)	1.92^*∗∗∗*^	0.51 (0.09~0.92)	1.66^*∗*^	0.37 (−0.04~0.79)	1.45

Patient	Warmness in hands	0.36 (0.01~0.71)	1.44^*∗*^	1.01 (0.66~1.37)	2.76^*∗∗∗*^	0.97 (0.52~1.43)	2.65^*∗∗∗*^	0.99 (0.53~1.45)	2.69^*∗∗∗*^
	Warmness in feet	0.29 (−0.07~0.65)	1.34	1.21 (0.85~1.57)	3.36^*∗∗∗*^	0.68 (0.21~1.15)	1.98^*∗∗∗*^	0.75 (0.27~1.23)	2.11^*∗∗*^
	Warmness in abdomen	0.22 (−0.14~0.58)	1.25	0.98 (0.61~1.34)	2.66^*∗∗∗*^	0.14 (−0.31~0.59)	1.15	0.11 (−0.35~0.57)	1.12
	Digestive ability	0.52 (0.16~0.88)	1.69^*∗∗*^	0.28 (−0.08~0.64)	1.32	−0.07 (−0.53~0.38)	0.93	0.06 (−0.39~0.52)	1.07
	Appetite	0.27 (−0.10~0.64)	1.31	0.33 (−0.05~0.71)	1.39	0.87 (0.41~1.33)	2.39^*∗∗∗*^	0.52 (0.06~0.97)	1.68^*∗*^
	Food portion	0.14 (−0.22~0.50)	1.15	0.29 (−0.07~0.65)	1.34	0.93 (0.47~1.39)	2.53^*∗∗∗*^	0.66 (0.19~1.12)	1.93^*∗∗*^
	Eating pace	0.05 (−0.31~0.40)	1.05	0.44 (0.08~0.81)	1.56^*∗*^	1.03 (0.58~1.47)	2.79^*∗∗∗*^	1.01 (0.56~1.46)	2.73^*∗∗∗*^

Base category: CG, females, SE type; ^*∗*^*p* < 0.05; ^*∗∗*^*p* < 0.01, ^*∗∗∗*^*p* < 0.001.
